# Supplementary material for: Microbial abundance, activity and population genomic profiling with mOTUs2
Source: Nat Commun. 2019 Mar 4;10:1014. doi: 10.1038/s41467-019-08844-4 (PMC6399450; doi:10.1038/s41467-019-08844-4)
Supplement: Supplementary file 1 — Supplementary Information [file 41467_2019_8844_MOESM1_ESM.pdf]

nature COMMUNICATIONS

Supplementary Information

Microbial abundance, activity, and population genomic profiling with mOTUs2

Alessio Milanese[1,*], Daniel R Mende[2,*], Lucas Paoli[3,4], Guillem Salazar[3], Hans-Joachim Ruscheweyh[3], Miguelangel Cuenca[3], Pascal Hingamp[5], Renato Alves[1,6] , Paul I Costea[1], Luis Pedro Coelho[1], Thomas S B Schmidt[1], Alexandre Almeida[7,8], Alex L Mitchell[7], Robert D Finn[7], Jaime Huerta-Cepas[1,9], Peer Bork[1,10,11,12], Georg Zeller[1,#], Shinichi Sunagawa[3,#]

[1] European Molecular Biology Laboratory, Heidelberg, Germany
[2] Daniel K. Inouye Center for Microbial Oceanography Research and Education, University of Hawai'i at Manoa, Honolulu, USA
[3] Department of Biology and Swiss Institute of Bioinformatics, ETH Zurich, Zurich, Switzerland
[4] Department of Biology, École normale supérieure, Paris, France
[5] Aix Marseille Univ, Université de Toulon, CNRS, IRD, MIO UM 110, 13288, Marseille, France
[6] Candidate for Joint PhD degree from EMBL and Heidelberg University, Faculty of Biosciences
[7] European Molecular Biology Laboratory, European Bioinformatics Institute (EMBL-EBI), Wellcome Genome Campus, Hinxton, United Kingdom
[8] Wellcome Trust Sanger Institute, Wellcome Genome Campus, Hinxton, United Kingdom
[9] Centro de Biotecnología y Genómica de Plantas, Universidad Politécnica de Madrid (UPM) - Instituto Nacional de Investigación y Tecnología Agraria y Alimentaria (INIA), Campus de Montegancedo-UPM, 28223-Pozuelo de Alarcón (Madrid) Spain
[10] Max Delbrück Centre for Molecular Medicine, Berlin, Germany
[11] Molecular Medicine Partnership Unit, Heidelberg, Germany
[12] Department of Bioinformatics, Biocenter, University of Würzburg

* contributed equally
# corresponding authors: zeller@embl.de, ssunagawa@ethz.ch

Supplementary Figures

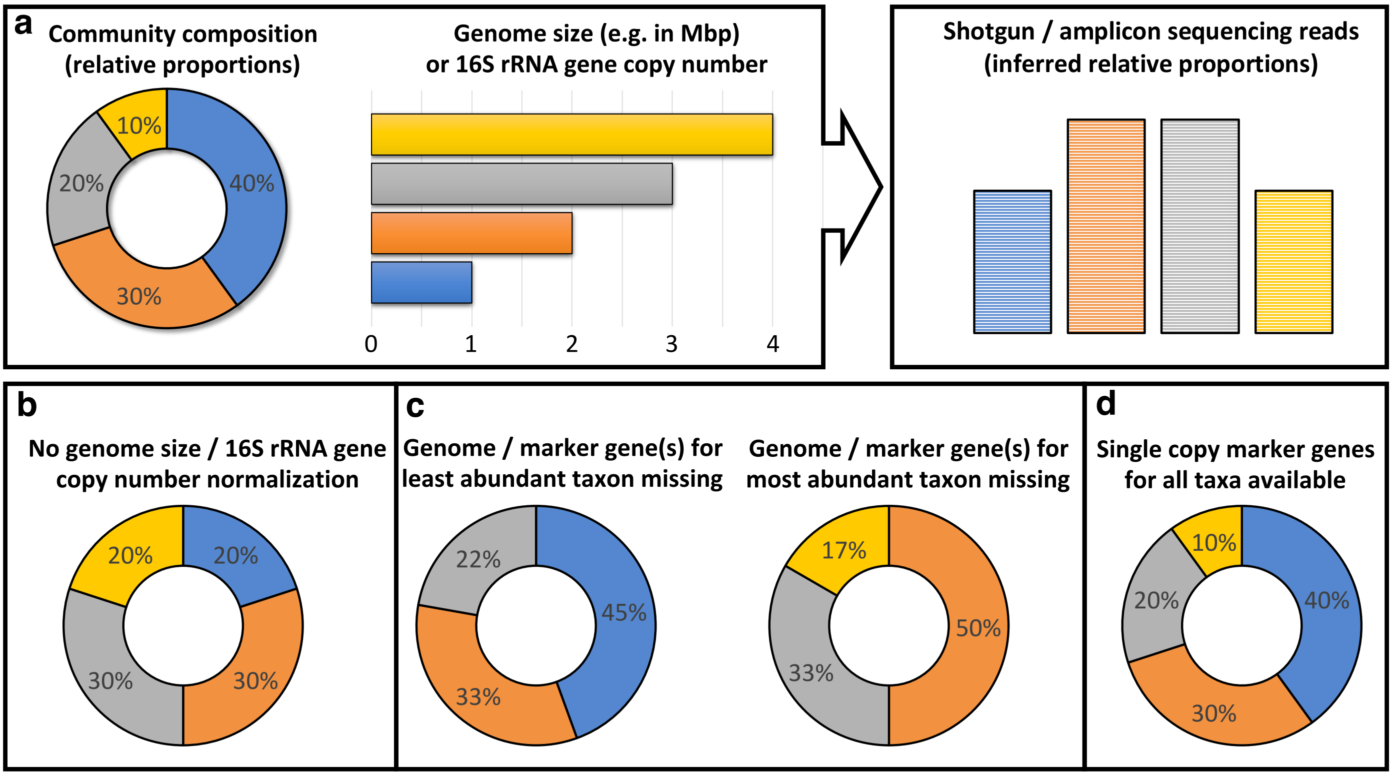

Supplementary Figure 1 | Variations in marker gene copy number, missing genome size normalization and missing taxa in reference data bases as major sources of biases in microbial community composition profiling. A conceptual example illustrates that random shotgun sequencing of a microbial community of species with different genomes sizes (**a**) will result in a distribution of sequencing reads that corresponds to the product of the relative proportion and genome size of each individual member (**b**). To infer the relative proportions of community members from this pool of shotgun sequencing reads, read abundances need to be normalized by estimated genome sizes. As an alternative, read abundances of clade-specific marker genes can be used, if they are known to occur only once per genome. Both approaches depend on prior knowledge and may introduce biases if unknown species are not detected (**c**). Ideally, single copy marker genes are available for both known and unknown species to resolve these biases (**d**). Other technical biases that may arise from sample processing including DNA extraction, sequencing library preparation, and bioinformatics data processing are illustrated in Nayfach and Pollard (2016)[1].

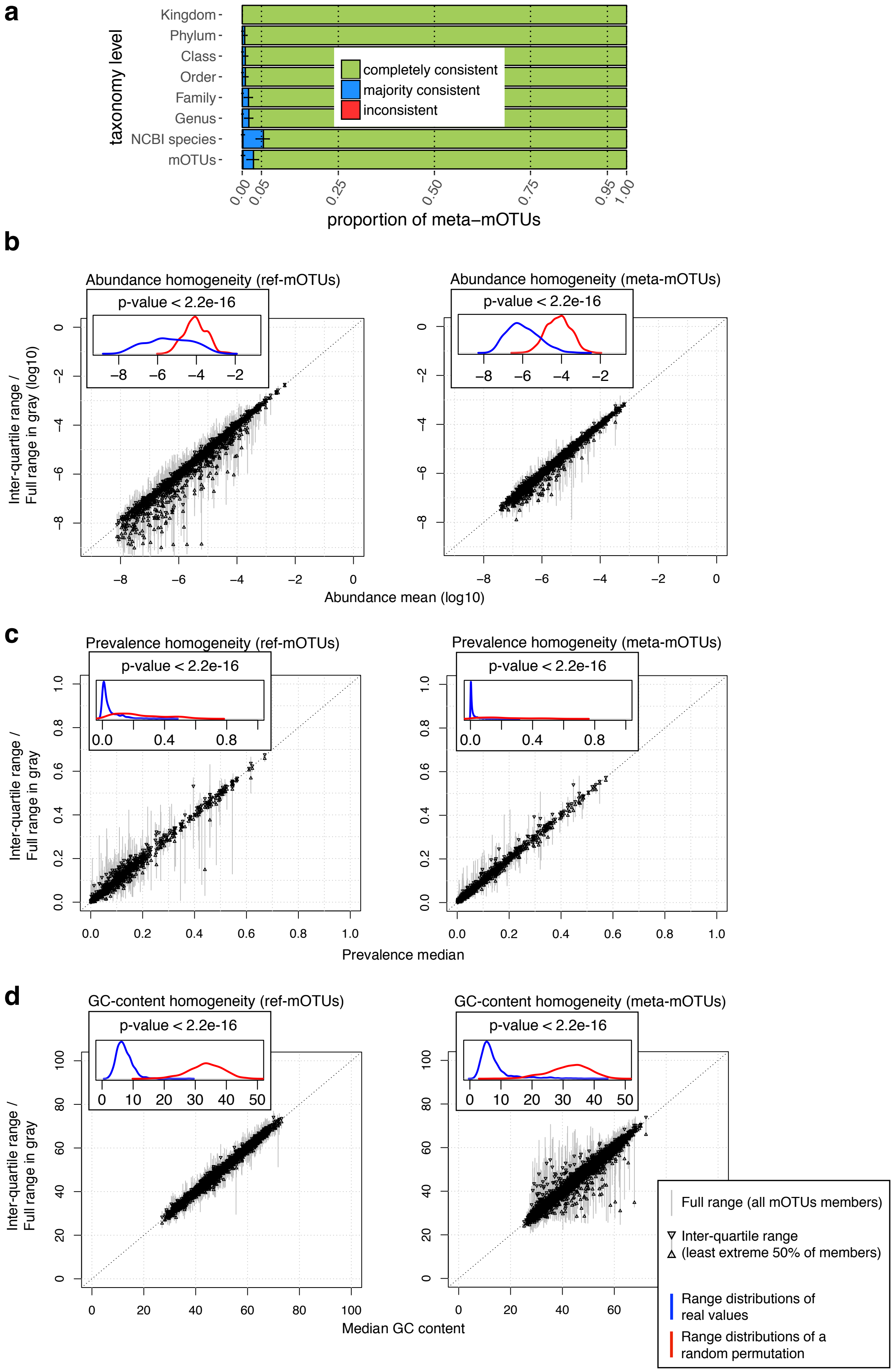

Supplementary Figure 2 | External evaluation of meta-mOTUs. (**a**) Expected error in linking the meta-mOTUs based on cross-validation of ref-mOTUs (see Methods). "mOTUs" refers to the MG-based grouping for the ref-mOTUs, which is different from NCBI species definitions. Values represent means and error bars denote standard deviations. (**b,c,d**) Homogeneity of relative abundance, prevalence and GC content broken down by ref- and meta-mOTUs. Inset boxes show the full range distribution of the observed values (blue) and of a random permutation (red). All distributions are significantly different (Wilcoxon test).

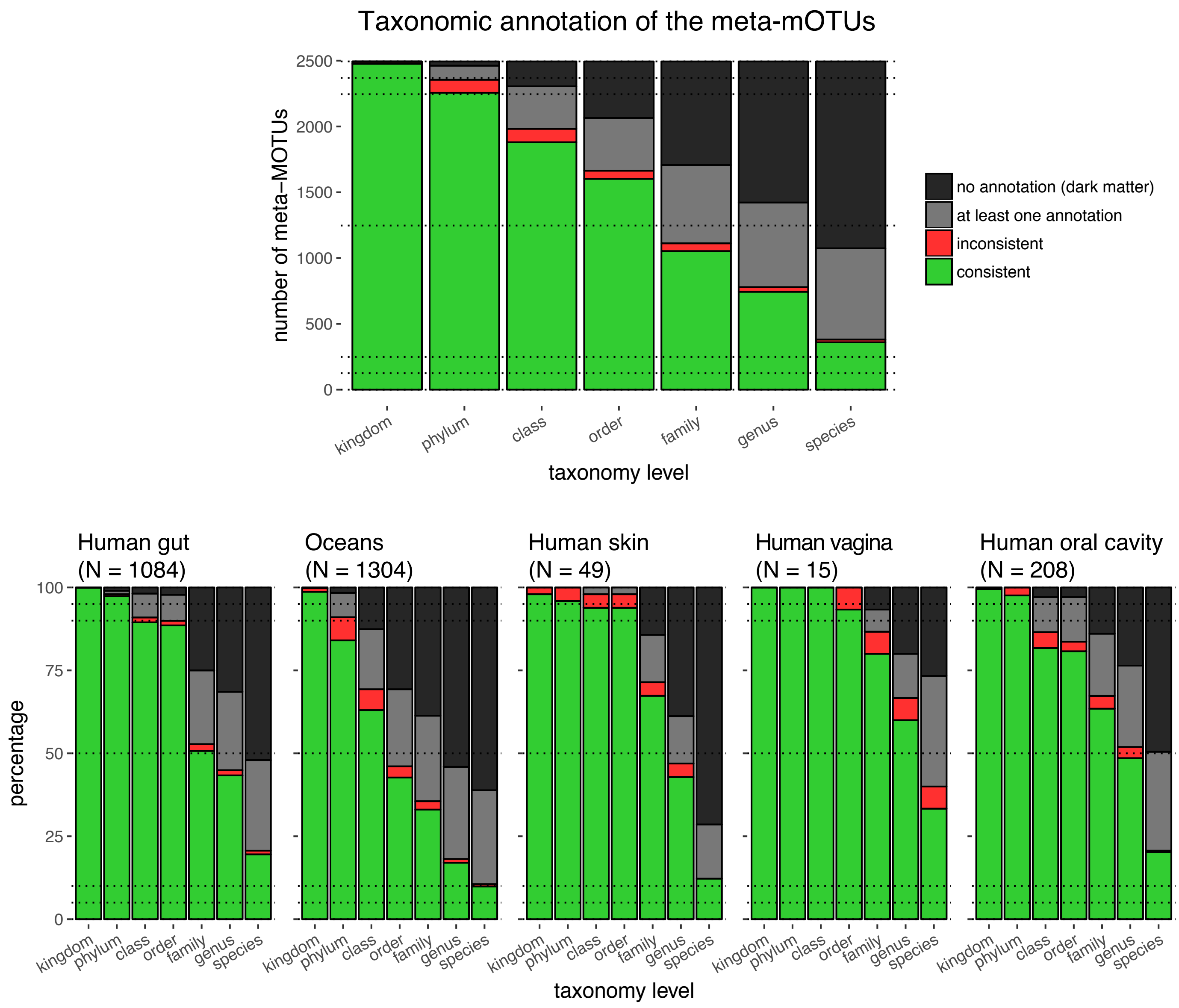

Supplementary Figure 3 | Taxonomic annotation of meta-mOTUs for seven major taxonomic ranks. Every marker gene was annotated with UniRef by a last common ancestor approach (see Methods). Based on the congruency and information of the marker genes within a mOTU, it was possible to decide between: no annotation (no annotation for any marker gene), at least one annotation (there is marker gene information for one or two genes), inconsistent (there is marker gene information for at least three genes and less than 50% are congruent), consistent (there is marker gene information for at least three genes and more than 50% are congruent). The top panel represents all 2,494 mOTUs, while the lower panels represent a breakdown by biome.
N = number of meta-mOTUs annotated in the specific biome

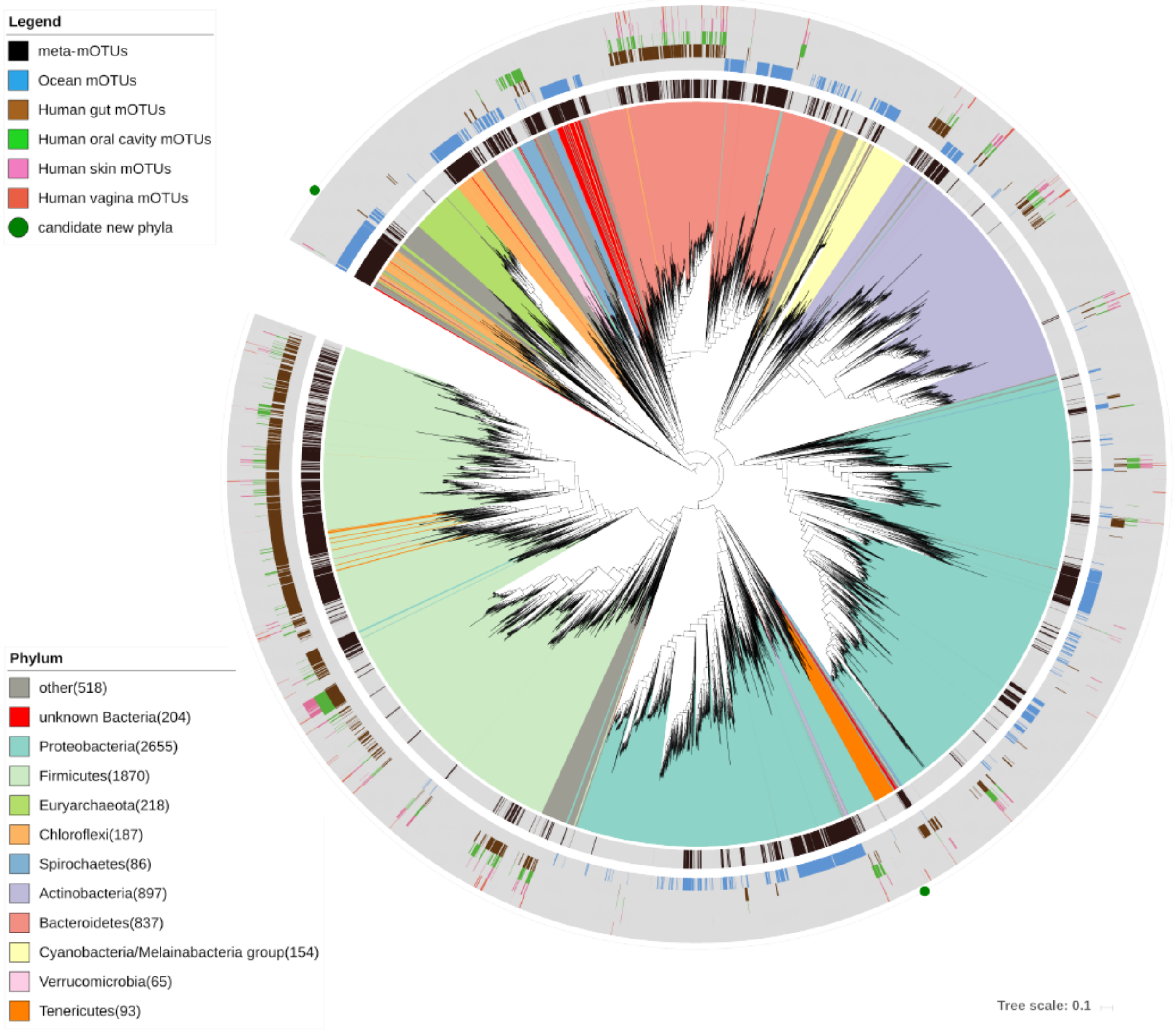

Supplementary Figure 4 | Phylogenetic tree constructed using the ten marker genes (see Methods). The internal colours represent the annotation of the ten most abundant phyla (plus grey for less abundant phyla and red for meta-mOTUs that lack phylum level annotations). The first outer circle represents the position of meta-mOTUs in black. The meta-mOTUs are spread across the tree, even if there are some hot spots of new prokaryotic species. The next circle represents environmental information of the mOTUs across 2,481 metagenomic samples (note that 4,315 ref-mOTUs do not have biome information because they were not detected in any sample). The outermost circle shows the position of two meta-mOTUs (meta_mOTU_v2_6300 and meta_mOTU_v2_6477) that map to MAGs that were recently proposed to represent the first genomic representative of their respective phyla (UAP2 and UBP8: Parks, Nature Microbiology, 2017)[2].

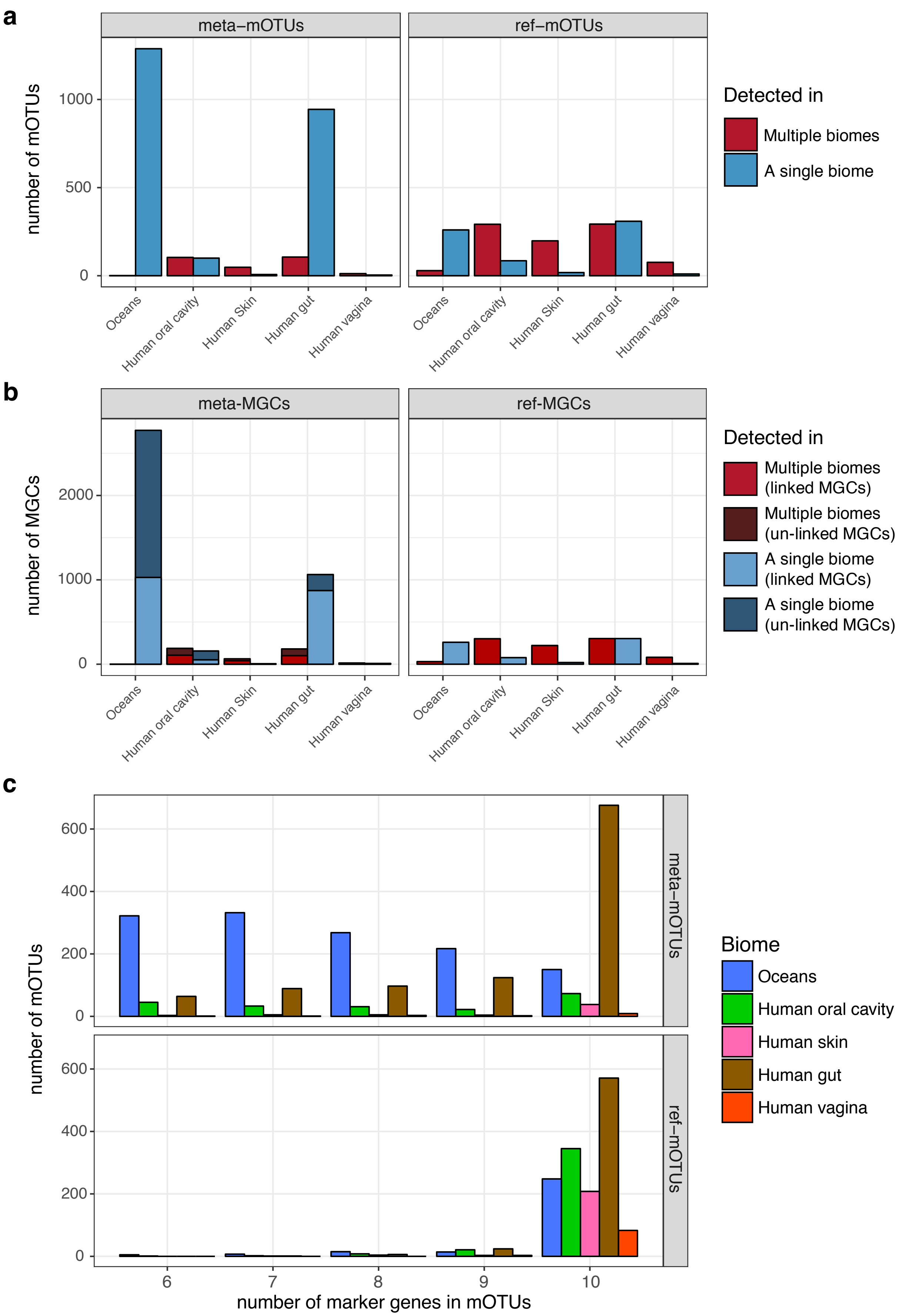

Supplementary Figure 5 | Biome information for mOTUs. (**a**) Number of mOTUs detected in the five studied biomes (see Methods), split by mOTU type (ref-mOTU and meta-mOTU). mOTUs detected in multiple environments are shown in red. Compared to the meta-mOTUs, the ref-mOTUs appear to be shared between more biomes, possibly reflecting the fact that are easier to cultivate. (**b**) Same display as (**a**), but on COG0012 MGCs only, showing that the observed biome-specificity is independent of the mOTU linking. (**c**) Number of marker genes that comprise the mOTUs. While for the ref-mOTUs it is frequently possible to detect all ten marker genes (MGs), for meta-mOTUs the linking of genes is more difficult (in particular for ocean-related mOTUs).

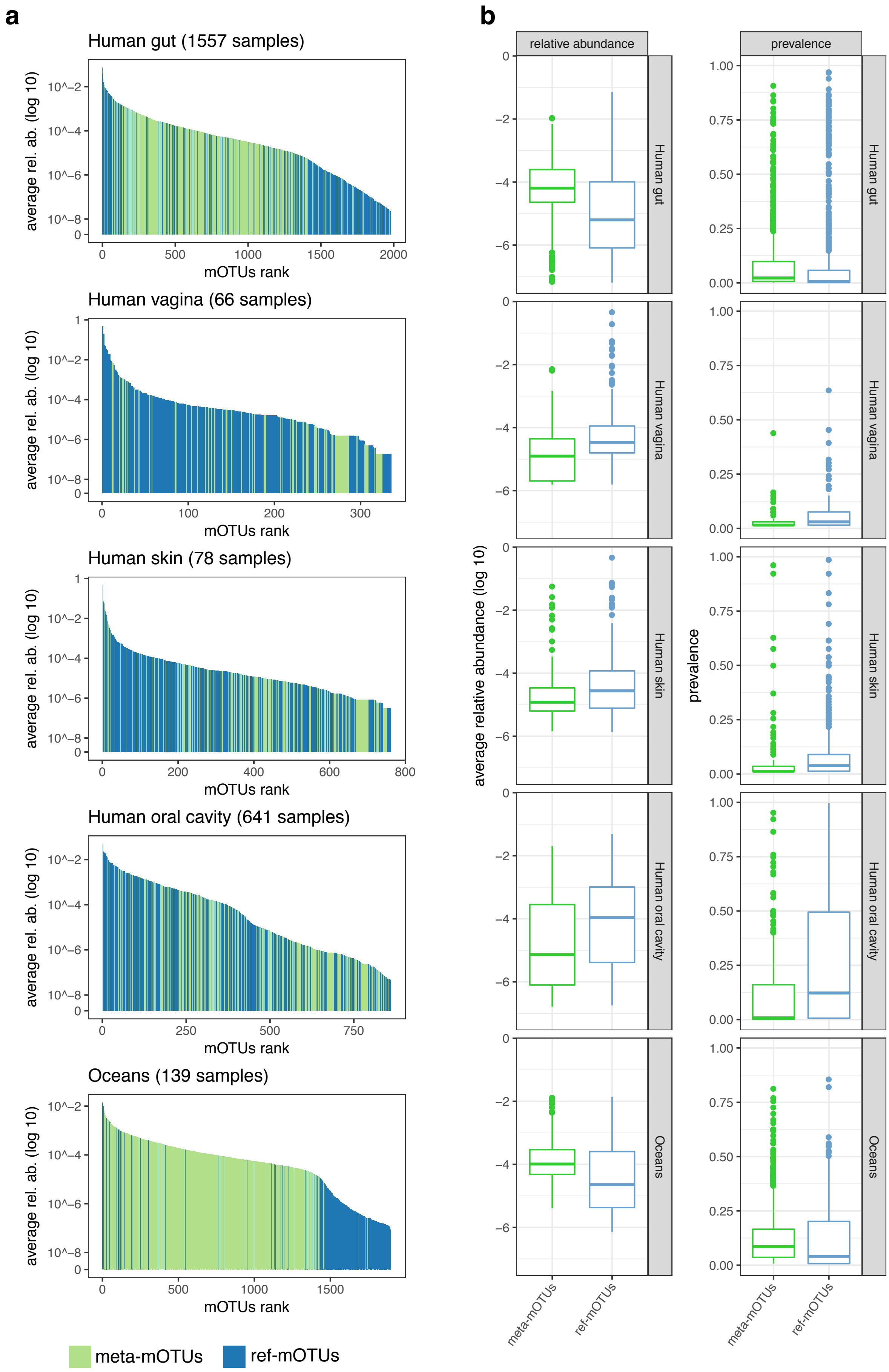

Supplementary Figure 6 | Observed richness and relative abundance of ref-mOTUs and meta-mOTUs on 2,481 metagenomic samples (Methods). (**a**) Rank abundance curves per biome. (**b**) Average relative abundance and prevalence (determined by rel. abundances > 10e-4) for meta-mOTUs and ref-mOTUs.

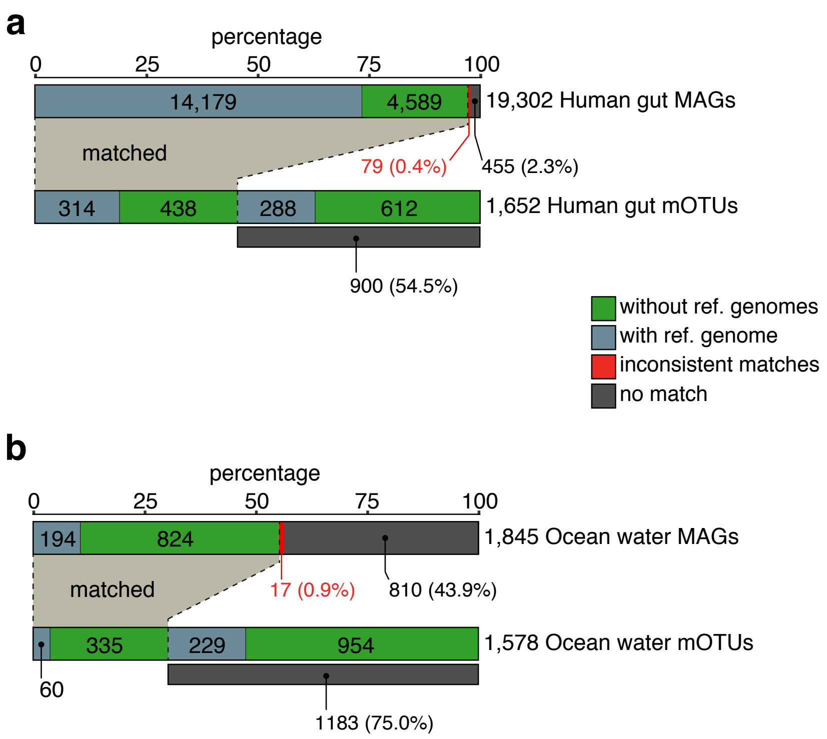

Supplementary Figure 7 | Comparison of metagenome-assembled genomes (MAGs) to mOTUs (see Methods). For human gut samples (**a**) mOTUs capture almost all (>97%) of the MAGs, while the MAGs cover only 45.5% of the mOTUs. For ocean water samples (**b**) both methods contain species that cannot be detected with the other method, reflecting what is represented in Fig. 1b. MAGs and mOTUs show high agreement (less than 1% of the MAGs is inconsistent with the mOTUs): this represents an additional external validation of the meta-mOTUs.

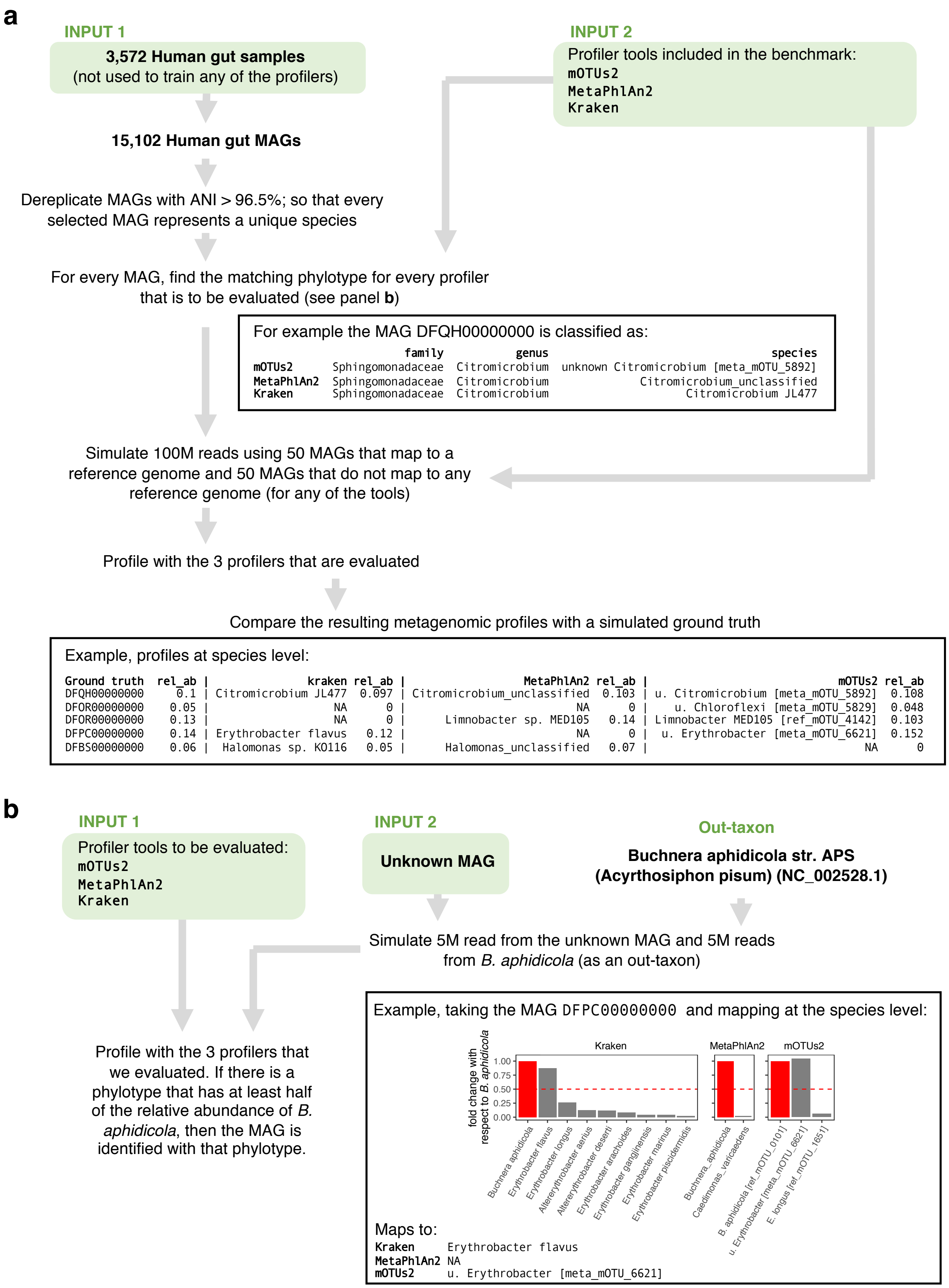

Supplementary Figure 8 | Simulation and benchmarking of the human gut metagenomic samples. (**a**) To be able to assess taxonomic quantification accuracy, human gut metagenomes were simulated using 15,102 human gut MAGs (see Methods). (**b**) To establish correspondence between MAGs and phylotypes quantified by the respective metagenomic profiler in an impartial way, for each MAG we simulated a test sample using reads from only that MAG to test how these were classified, that is, which taxonomic entity each profiler assigned to the MAG. In order to avoid spurious classifications due to non-specific low-abundance phylotypes, we also added reads from a non-gut microbial genome (*Buchnera aphidicola*) as an out-taxon to the test samples to be able to compare the relative abundance; in cases where the relative abundance of the phylotype assigned to the MAG of interest (i.e., the most abundant one) was less than half that for *B. aphidicola*, we concluded that this MAG was likely not represented in the reference database of that tool (as shown for MetaPhlAn2 in the example).

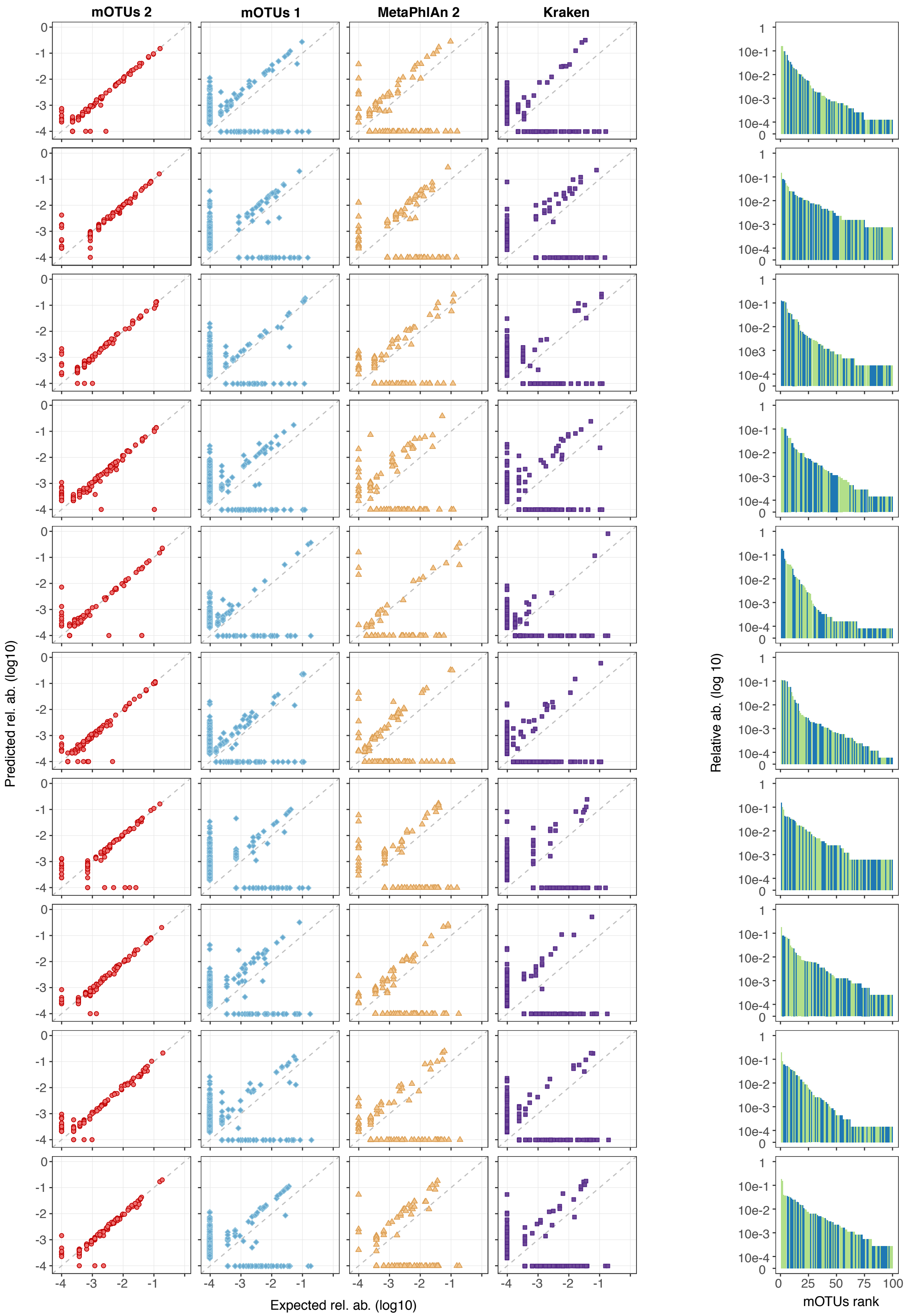

Supplementary Figure 9 | Benchmarking species quantification accuracy on ten simulated metagenomic samples. Each sample contains 50 MAGs with and 50 MAGs without a representative reference genome sequence (Methods). Every row in the graph corresponds to a sample. The first four columns represent the profiles generated with mOTUs2, mOTUs1, MetaPhlAn2 and Kraken (red, blue, yellow and purple, respectively); the fifth column represents the rank abundance curves of the simulated species (in blue MAGs with a representative genome and in green MAGs without a representative genome).

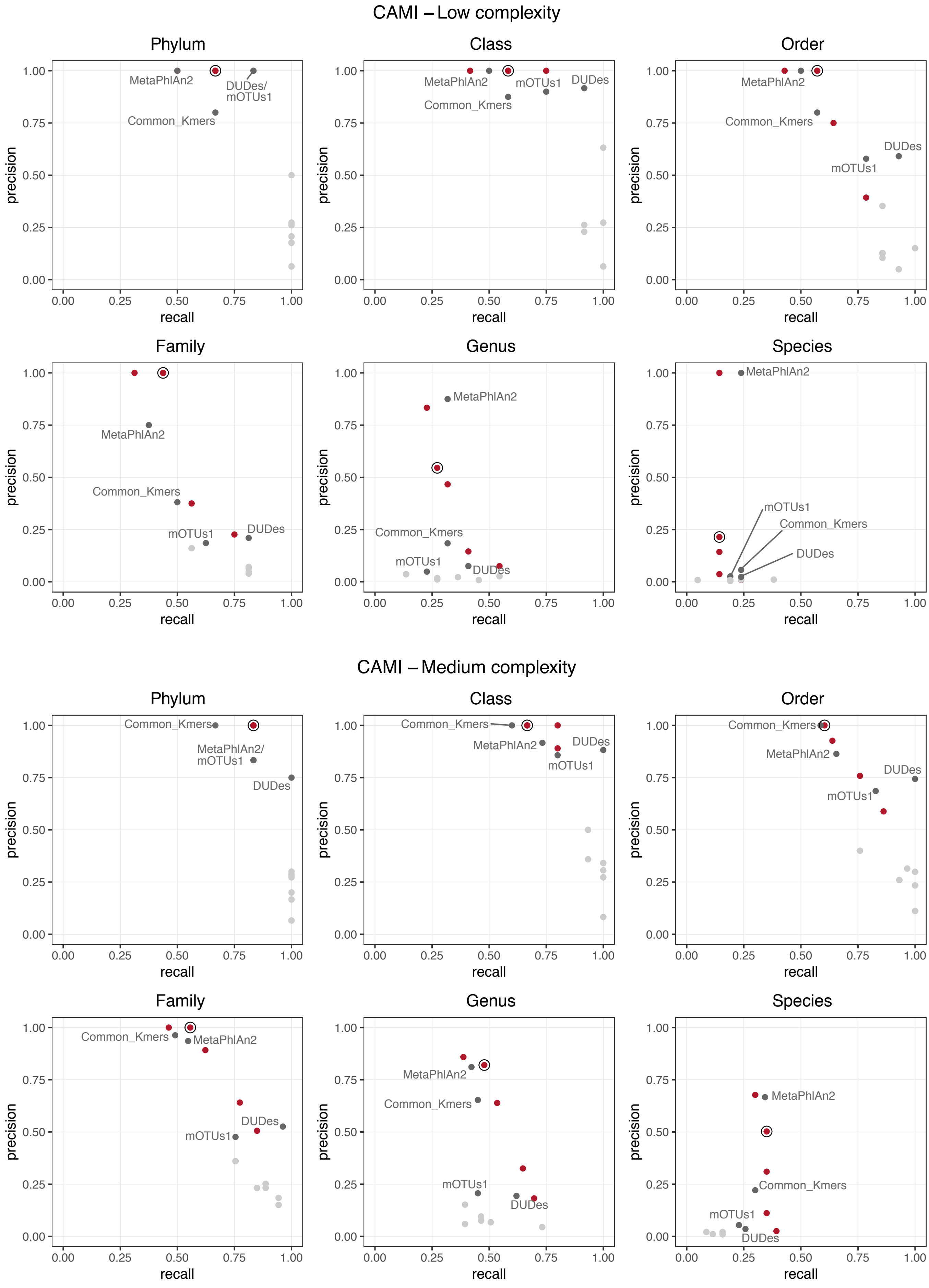

Supplementary Figure 10 – PART 1 | Evaluation of precision and recall on the CAMI dataset. (Figure legend on the following page).

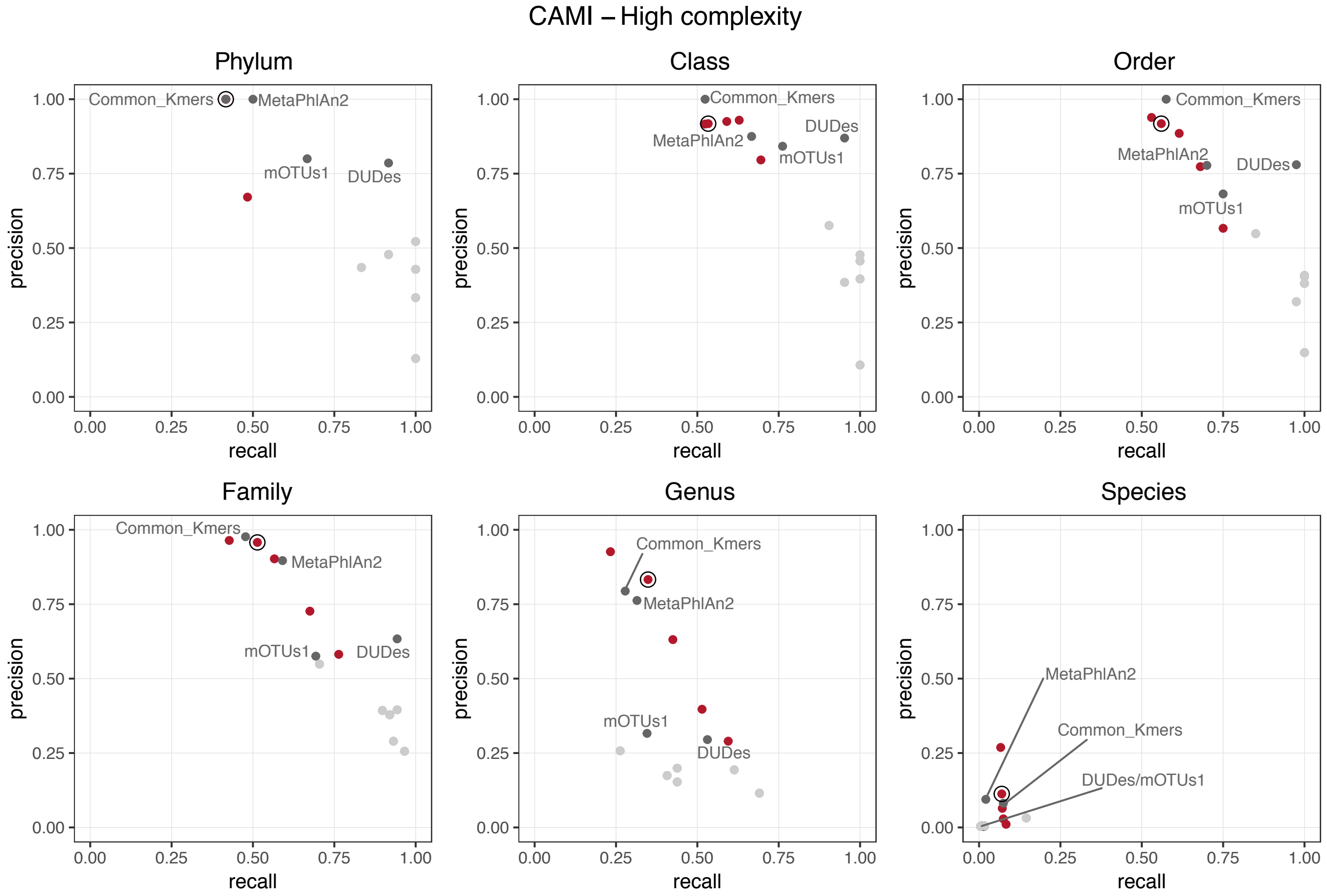

Supplementary Figure 10 – PART 2 | Evaluation of precision and recall on the CAMI dataset[3]. For the medium and high complexity datasets (Sczyrba et al., 2017)[3], plotted values are the average of two and five samples respectively. The results of mOTUs2 with five different parameter settings are shown in red (high precision (-l 140 -g 6), default (-l 100 -g 3), recall (-l 75 -g 3), high recall (-l 50 -g 2) and highest recall (-l 30 -g 1), see Methods); the red dot with a black circle represents the result obtained with default parameters. We represent in dark grey the four profilers with an average precision greater than 0.5 (see labels), in light grey six other profilers evaluated by CAMI that have average precision lower than 0.5 (MetaPhyler, TIPP, Taxy.Pro, FOCUS, CLARK and Quickr).

a

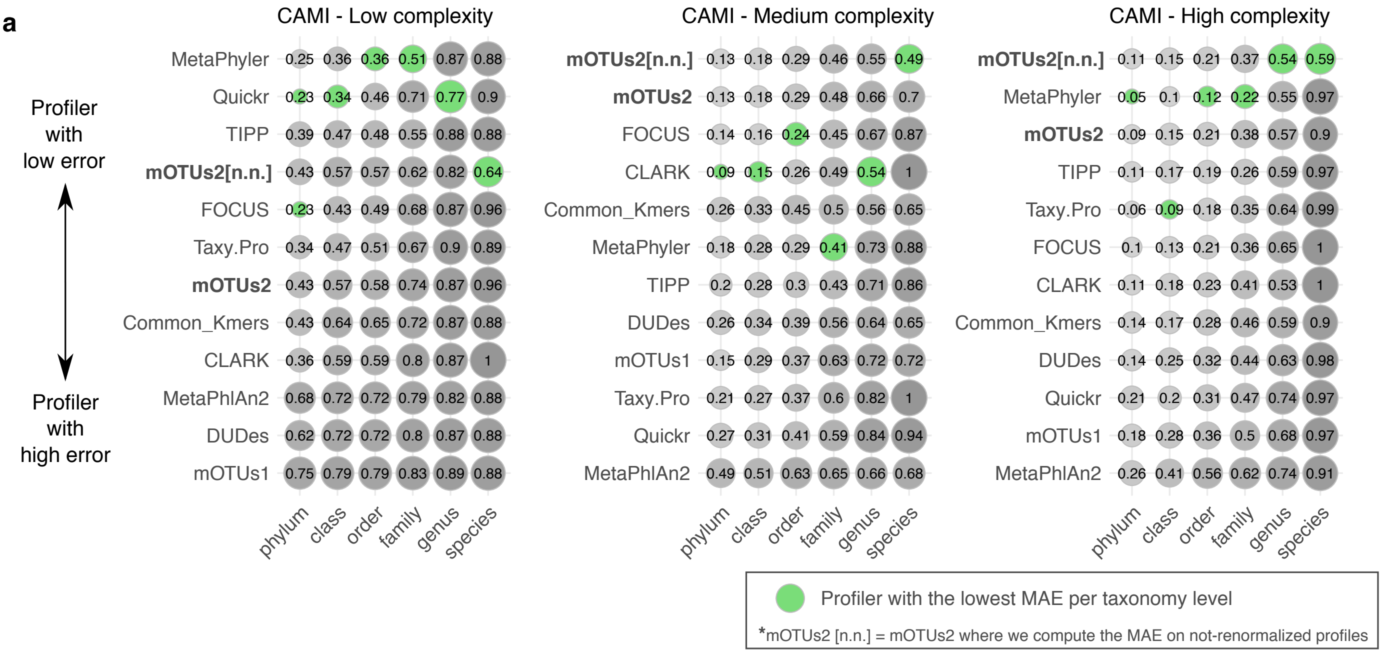

b

taxa	gold	profiled	re-normalized
species 1	0.3	0	0
species 2	0.25	0.25	0.5
species 3	0.2	0	0
species 4	0.15	0.15	0.3
species 5	0.1	0.1	0.2
(unmapped)		(0.5)	

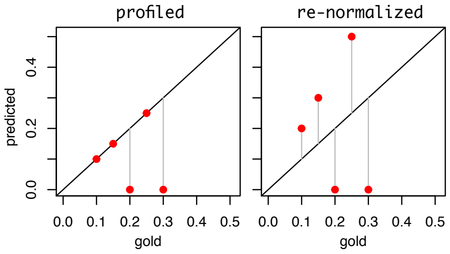


```
       MAE (gold, profiled) = |0.3 - 0|+|0.25 - 0.25|+|0.2 - 0|+|0.15 - 0.15|+|0.1 - 0.1|
                            =     0.3  +      0      +    0.2  +      0      +     0
                            = 0.5
MAE (gold, re-normalized) = |0.3 - 0|+|0.5  - 0.25|+|0.2 - 0|+|0.3  - 0.15|+|0.2 - 0.1|
                            =     0.3  +     0.25     +    0.2  +     0.15     +    0.1
                            = 1.0
```

Supplementary Figure 11 | Evaluation of the mean absolute error (MAE, called L1 in CAMI) in the CAMI challenge[3]. (**a**) Representation of the MAE for ten profilers evaluated in the CAMI challenge and mOTUs2. Note that the MAE is calculated on re-normalised data (see below) for all rows except "mOTUs2 [n.n.]" (not re-normalised]). The profilers are ordered by best performing (top) to worst (bottom), based on the average position per taxonomic rank. The profiler with the lowest MAE on a given taxonomic rank is highlighted in green. All values for mOTUs2 were calculated with the OPAL package (version 0.2.9 from https://github.com/CAMI-challenge/OPAL) which was developed from the CAMI challenge. (**b**) A toy example that explains how the benchmark can be distorted by calculating the MAE on re-normalised data. If the profiled abundances are re-normalised, relative abundances become distorted, that is, profiled taxa are over-estimated, whereas mOTUs (both the first and second version) estimates the unmapped fraction of reads using unbinned MGCs (see Methods).

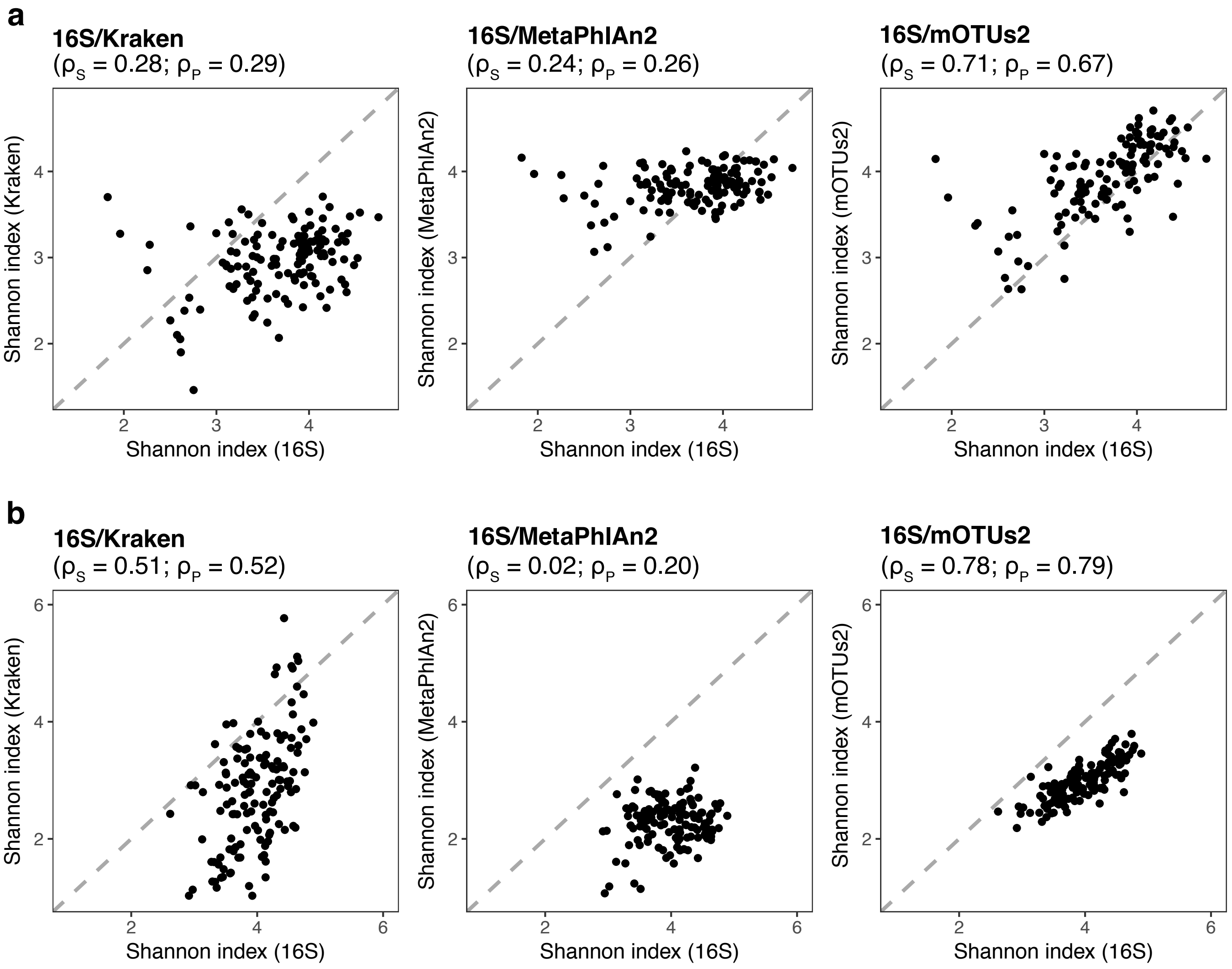

Supplementary Figure 12 | Microbial community diversity estimates compared between three tools (Kraken, MetaPhlan2 and mOTUs2). The computed values are compared to diversity estimates calculated based on 16S rRNA gene (16S) profiles for 129 human fecal samples (**a**) and 139 ocean water samples (**b**) generated from the very same DNA extracts as shotgun metagenomic data used as input to the profiling tools. See Methods for details.
ρ_S - Spearman correlation, ρ_P - Pearson correlation.

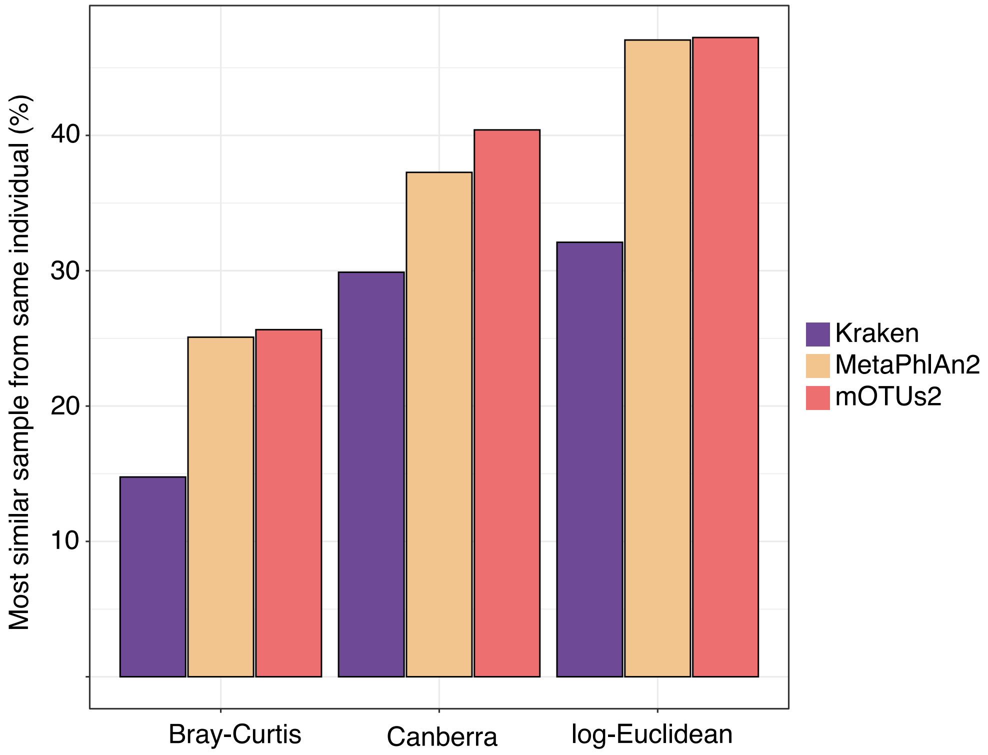

Supplementary Figure 13 | Percentage of samples for which the most similar community composition profile matches another sample from the same individual from the same body site. Bray-Curtis, Canberra and log-Euclidean distances were calculated for all pairs of samples from the Human Microbiome Project[4]. For each sample, we evaluated if the most similar sample originated from the same or from a different individual.

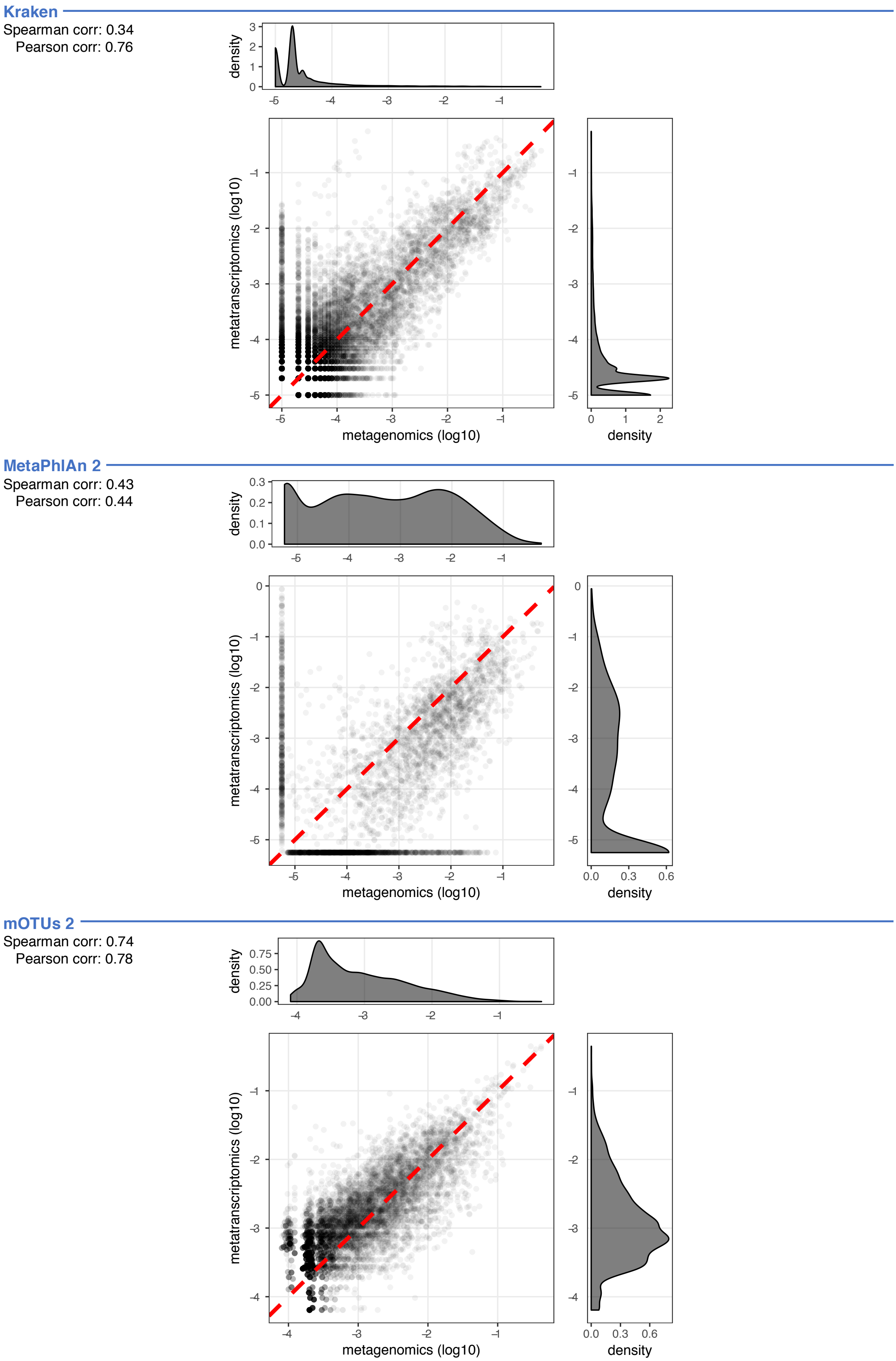

Supplementary Figure 14 | Correlation between metagenomic and metatranscriptomic profiles compared between profiling methods. Every dot in the scatter plot represents a species in one sample (N = 36 samples from Heintz-Buschart et al.[5]). See Methods for a description of how samples were processed.

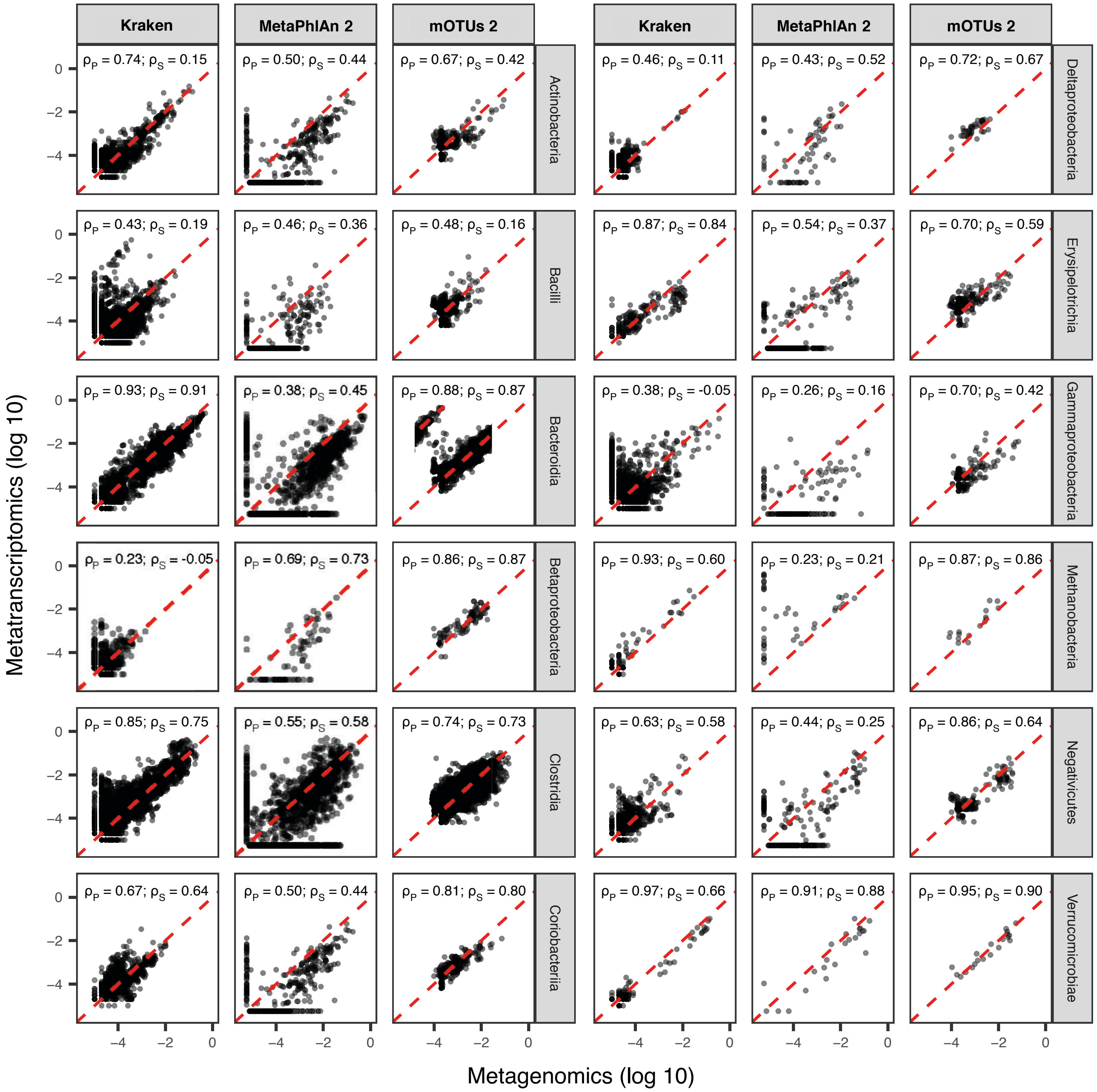

Supplementary Figure 15 | Breakdown of metagenomic versus metatranscriptomic profile correlations by taxonomic rank of class and method for 36 fecal samples. Metagenomic and metatranscriptomics profiles are matched (i.e. produced from the same sample); see Methods for details.

ρ_S - Spearman correlation, ρ_P - Pearson correlation.

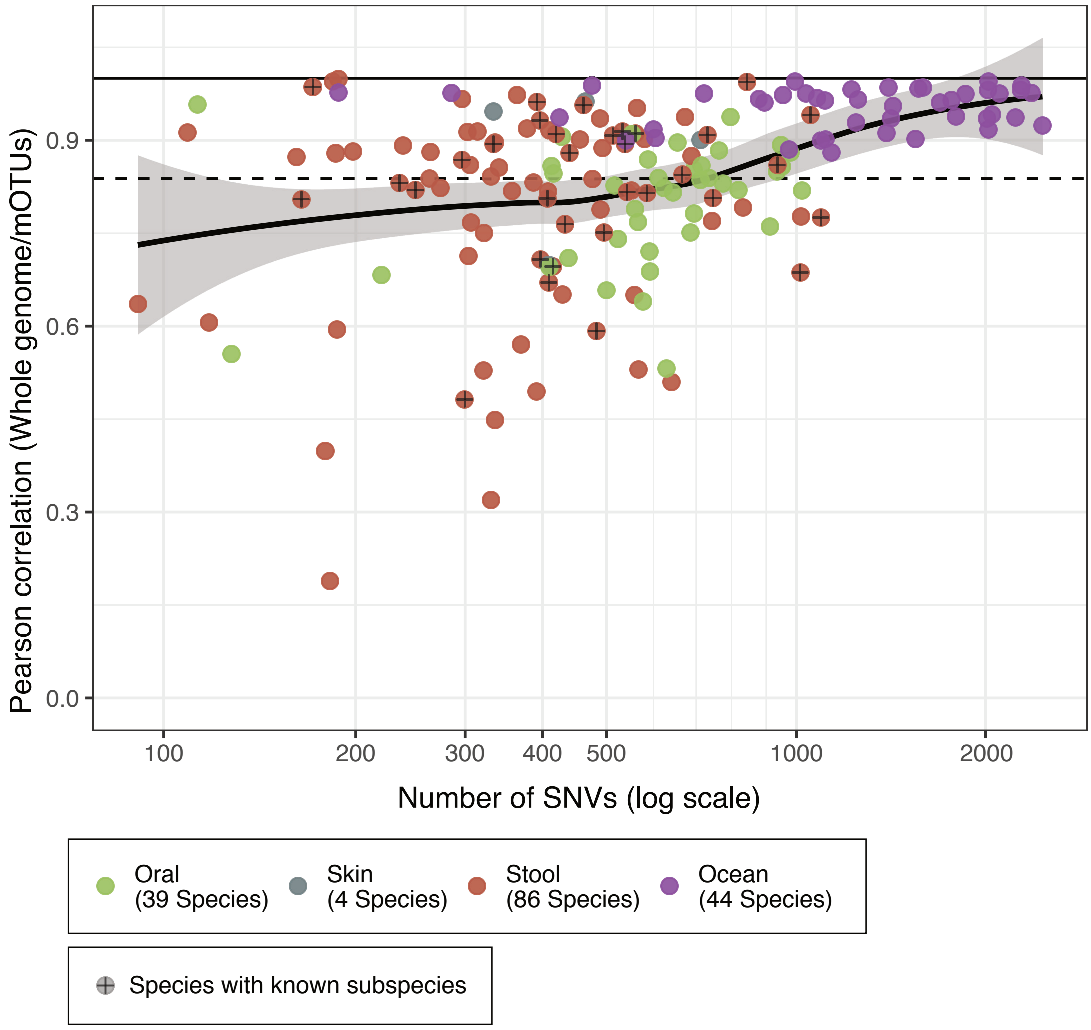

Supplementary Figure 16 | Correlation coefficient between SNV profiles generated using MGs and whole genome as a function of the number of SNVs identified on the MGs across all samples for a given mOTUs (log scale). Each data point represents the Pearson correlation of two SNV profiles for a given species. The correlations are computed between genetic distances based on whole genomes (ProGenomes, Mende et al., NAR, 2016)[6] or universal single copy marker genes (MGs) as a reference for deriving SNV profiles across all matching samples passing metaSNV filtering steps (see Methods). The correlations are represented as a function of the number of SNVs identified across the marker genes of a given species. Crossed dots represent ProGenomes genomes for which subspecies have been identified (Costea et al., MSB, 2017)[7].

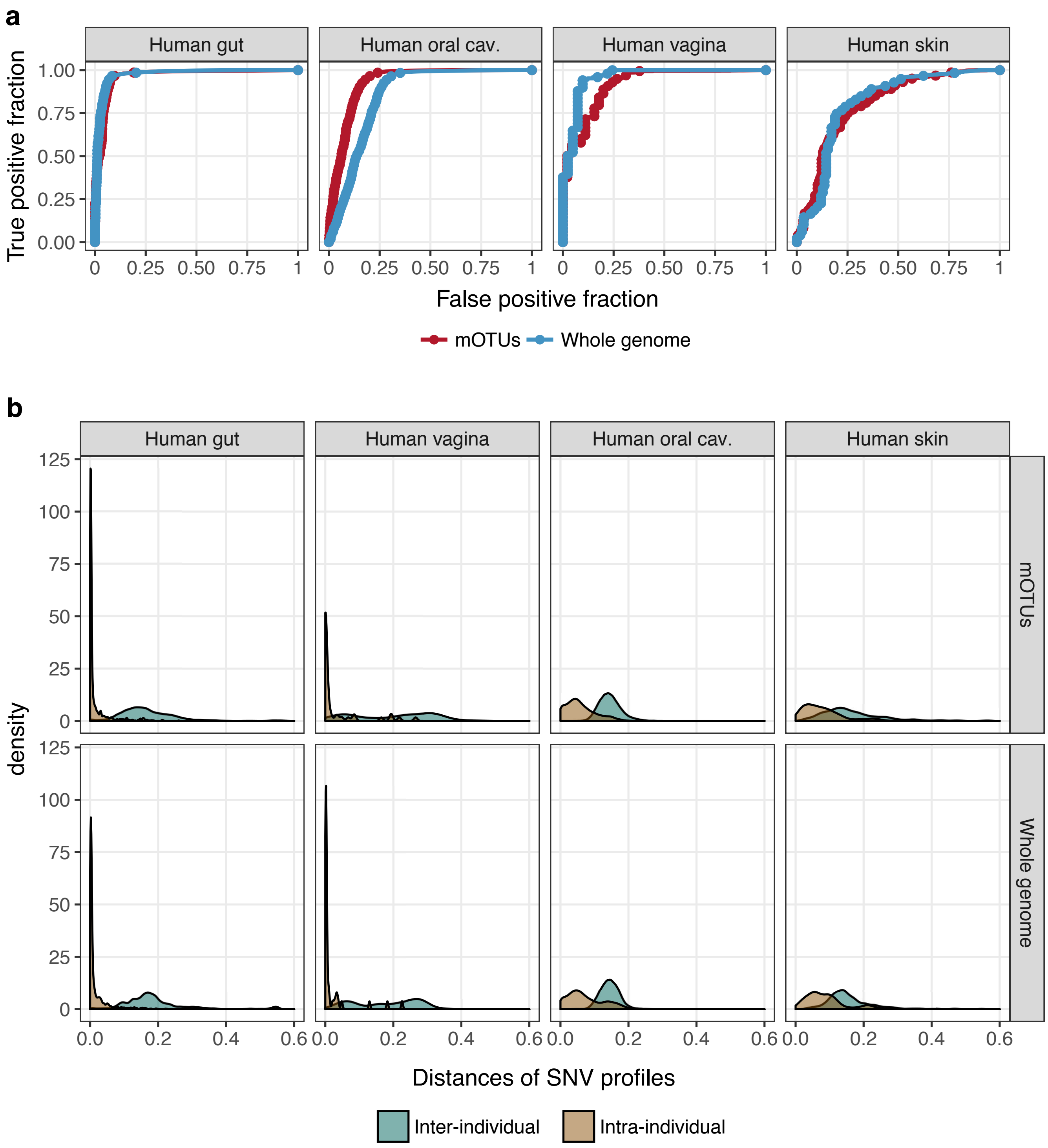

Supplementary Figure 17 | Consistency of intra- versus inter-individual population genomic distance estimates when using MGs or whole genomes for SNV profiling of HMP samples from different body sites.
(**a**) Receiver operating characteristic (ROC) curves for intra-individual specificity of whole genome and MGs based genetic distances. True positives correspond to cases where the greatest intra-individual genetic distance is smaller than the smallest inter-individual genetic distance, whereas false positives correspond to cases where the smallest inter-individual distance is smaller. These values are computed for each combination of individual/species (for both MGs and whole genomes) where enough samples passed metaSNV[8] filtering steps (see Methods). (**b**) Density plot for the intra- and inter-individual genetic distances used to compute the ROC curves. The density is computed across all combinations of individual/species for both MGs (mOTUs) and whole genomes.

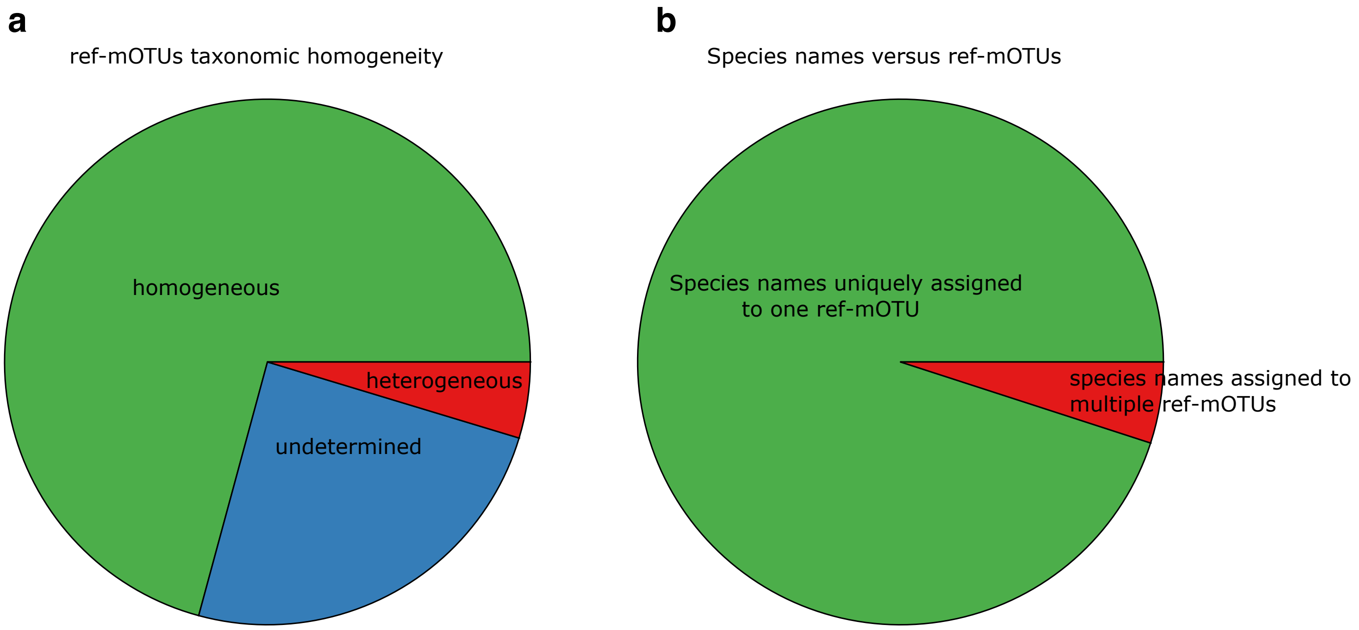

Supplementary Figure 18 | Congruence between NCBI taxonomy and ref-mOTUs.
(**a**) Taxonomic consistency of ref-mOTUs (homogeneous clusters in green; heterogeneous clusters in red; undetermined clusters (containing only genomes with non-binomial names) in blue). (**b**) Distribution of species names among ref-mOTUs (species names uniquely assigned to one ref-mOTU in green; species names assigned to multiple ref-mOTUs in red).

Supplementary Tables

	Method \ Dataset	**Log datasets** Average	S.d.
Spearman Correlation	mOTUs2	0.63	0.29
	MetaPhlAn2	**0.68**	0.11
	mOTUs1	0.3	0.19
	Kraken	0.22	0.16
	kraken+Braken	0.23	0.19
FALSE positives	mOTUs2	**10**	10
	MetaPhlAn2	13	7
	mOTUs1	13	10
	Kraken	20	12
	kraken+Braken	24	14
FALSE negative	mOTUs2	**21**	7
	MetaPhlAn2	33	10
	mOTUs1	33	13
	Kraken	33	15
	kraken+Braken	32	14

	Method \ Dataset	**Even datasets** Average	S.d.
Root mean square error	mOTUs2	0.82	0.24
	MetaPhlAn2	**0.34**	0.08
	mOTUs1	1.10	0.24
	Kraken	1.61	0.44
	kraken+Braken	0.9	0.25
FALSE positives	mOTUs2	12	12
	MetaPhlAn2	**10**	3
	mOTUs1	22	15
	Kraken	23	10
	kraken+Braken	29	11
FALSE negative	mOTUs2	**12**	11
	MetaPhlAn2	**12**	10
	mOTUs1	27	14
	Kraken	27	13
	kraken+Braken	23	11

Supplementary Table 1 | Performance achieved by mOTUs2 and the other metagenomic profilers evaluated on 22 simulated metagenomes from Truong *et al.* (2015)[9] (the table is adapted from Supplementary Table 6 of the same paper). The performance of MetaPhlAn2 is computed on archaea, bacteria, viruses and eukaryotes, whereas the other methods are scored on archaea and bacteria only. For MetaPhlAn2, mOTUs1 and Kraken, values from the original paper are shown. "Kraken+Bracken" corresponds to our analysis with Kraken (see Methods), whereas "Kraken" corresponds to the original evaluation by Truong *et al.* (2015)[9]; mOTUs2 was executed with default parameters. In order to map the 1,073 simulated prokaryotic genomes (for which only the name is provided), we first identified the NCBI taxonomy identifier and matched it to the reference genomes used to build the mOTUs (995 matches). For the remaining 81 genomes, 44 were matched at higher NCBI taxonomy level and 34 did not have a match in mOTUs. Note that these 34 genomes were excluded from the mOTU database because of low quality. Even the 995 "correct" matches contain some errors. For example, "Peptostreptococcaceae_noname Clostridium_difficile Clostridium_difficile_DA00310" simulated in the Even_10M_4 sample is mapped to ref_mOTU_v2_0643. However, when the sample is profiled with mOTUs2, it is classified as ref_mOTU_v2_0051 (also a *Clostridium difficile*), increasing both false positives and false negatives.

Supplementary References

1. Nayfach, S., Rodriguez-Mueller, B., Garud, N. & Pollard, K. S. An integrated metagenomics pipeline for strain profiling reveals novel patterns of bacterial transmission and biogeography. *Genome Res.* **26**, 1612-1625 (2016).
2. Parks, D. H.*, et al.* Recovery of nearly 8,000 metagenome-assembled genomes substantially expands the tree of life. *Nat. Microbiol.* **2**, 1533-1542 (2017).
3. Sczyrba, A.*, et al.* Critical Assessment of Metagenome Interpretation-a benchmark of metagenomics software. *Nat. Methods* **14**, 1063-1071 (2017).
4. Human Microbiome Project, C. Structure, function and diversity of the healthy human microbiome. *Nature* **486**, 207-214 (2012).
5. Heintz-Buschart, A.*, et al.* Integrated multi-omics of the human gut microbiome in a case study of familial type 1 diabetes. *Nat. Microbiol.* **2**, 16180 (2016).
6. Mende, D. R.*, et al.* proGenomes: a resource for consistent functional and taxonomic annotations of prokaryotic genomes. *Nucleic Acids Res.* **45**, D529-D534 (2017).
7. Costea, P. I.*, et al.* Subspecies in the global human gut microbiome. *Mol. Syst. Biol.* **13**, 960 (2017).
8. Costea, P. I.*, et al.* metaSNV: A tool for metagenomic strain level analysis. *PLoS One* **12**, e0182392 (2017).
9. Truong, D. T.*, et al.* MetaPhlAn2 for enhanced metagenomic taxonomic profiling. *Nat. Methods* **12**, 902-903 (2015).
