# Supplementary material for: Microbial abundance, activity and population genomic profiling with mOTUs2
Source: Nat Commun. 2019 Mar 4;10:1014. doi: 10.1038/s41467-019-08844-4 (PMC6399450; doi:10.1038/s41467-019-08844-4)
Supplement: Supplementary file 2 — Description of Additional Supplementary Files [file 41467_2019_8844_MOESM2_ESM.pdf]

Description of Additional Supplementary Files

Supplementary Data 1.
List of metagenomic samples used to create the meta-mOTUs.

Supplementary Data 2.
List of metagenomic samples used to create the Human Gut MAGs.

Supplementary Data 3.
Benchmarking quantification accuracy on ten simulated metagenomic samples (Methods). The values in the table are represented in Figure 2 a-g.

Supplementary Data 4.
Benchmarking quantification accuracy on eight simulated metagenomic samples from CAMI. The values in the table are represented in Figure 2 h-q and Supplementary Figure 10 and 11. The values for mOTUs2 are calculated with OPAL, all the other are the original values from the CAMI paper. There are five values for mOTUs2, which corresponds to five different set of parameters: high precision (-l 140 -g 6), default (-l 100 -g 3), recall (-l 75 -g 3), high recall (-l 50 -g 2) and highest recall (-l 30 -g 1).
